# Electrical brain activations in preadolescents during a probabilistic reward-learning task reflect cognitive processes and behavior strategies

**DOI:** 10.3389/fnhum.2025.1460584

**Published:** 2025-01-30

**Authors:** Yu Sun Chung, Berry van den Berg, Kenneth C. Roberts, Armen Bagdasarov, Marty G. Woldorff, Michael S. Gaffrey

**Affiliations:** ^1^Department of Psychology and Neuroscience, Duke University, Durham, NC, United States; ^2^Department of Psychology, Kean University, Union, NJ, United States; ^3^Experimental Psychology, University of Groningen, Groningen, Netherlands; ^4^Center for Cognitive Neuroscience, Departments of Psychiatry, Psychology and Neuroscience, and Neurobiology, Duke University, Durham, NC, United States; ^5^Children’s Wisconsin, Milwaukee, WI, United States; ^6^Division of Pediatric Psychology and Developmental Medicine, Department of Pediatrics, Medical College of Wisconsin, Milwaukee, WI, United States

**Keywords:** reinforcement learning, reward-related positivity, attention, N2pc, P300, win-stay-lose-switch strategy

## Abstract

Both adults and children learn through feedback to associate environmental events and choices with reward, a process known as reinforcement learning (RL). However, tasks to assess RL-related neurocognitive processes in children have been limited. This study validated a child version of the Probabilistic Reward Learning task in preadolescents (8–12 years) while recording event-related-potential (ERPs), focusing on: (1) reward-feedback sensitivity (frontal Reward-related Positivity, RewP), (2) late attention-related responses to feedback (parietal P300), and (3) attentional shifting toward favored stimuli (N2pc). Behaviorally, as expected, preadolescents could learn stimulus–reward outcome associations, but with varying performance levels. Poor learners showed greater RewP amplitudes compared to good learners. Learning strategies (i.e., Win-Lose-Stay-Shift) were reflected by feedback-elicited P300 amplitudes. Lastly, attention shifted toward to-be-chosen stimuli, as evidenced by the N2pc, but not toward more highly rewarded stimuli as in adults. These findings provide novel insights into the neural processes underlying RL in preadolescents.

## Introduction

From an early age, children learn about the world around them through trial and error. That is, through repeated experience, children progressively learn what actions are likely to result in relatively positive or negative outcomes given a specific environment and/or set of choices. The ability to learn associations between choice behavior and outcomes and to then change patterns of subsequent choice behavior, accordingly, has been called probabilistic reinforcement learning (PRL). Computational models of PRL often separate such learning into several sub-components associated with both behaviorally and neurobiologically distinguishable processes. Specifically, PRL models involve a sequence of hypothesized neurocognitive processes, including evaluation of the relative value associated with available choices, updating of stimulus–reward outcome associations based on feedback, and shaping of selective attention toward a more-likely-winning stimulus to guide future choice (Anderson, 2017). However, while some of the neural correlates of PRL have been well studied in adults, relatively few studies have investigated this question in adolescents or children (reviewed by DePasque and Galván, 2017).

This limited PRL research in children is a critical gap in knowledge, as studying PRL in children could be valuable for identifying specific neurocognitive processes that may account for individual differences in cognitive development, decision-making, and even risk-taking behavior (reviewed in Nussenbaum and Hartley, 2019). More importantly, neural mechanisms of PRL have been suggested as a promising risk factor for depression (reviewed in Kangas et al., 2022), particularly during the transitional period from late childhood to early adolescence (Hankin et al., 2015). Even asymptomatic adolescents of parents with depression have showed poor PRL behavior (Saulnier et al., 2023), which is thought to reflect impairments in the dopaminergic (DA) mesocorticolimbic regions (reviewed in Lerner et al., 2021). However, while many studies in developmental psychology and/or neuroscience focusing on adolescents or children emphasize reward-related behavior and developmental differences in neural responsivity to reward and punishment (Silverman et al., 2015), fewer studies have focused on key neural components of reward learning in the context of reinforcement learning (Nussenbaum and Hartley, 2019). The shortage of such research is partly due to the paucity of experimental tasks specifically designed for preadolescents. As a result, there has been limited research identifying the specific neurocognitive components of PRL that may be impaired in children at risk for depression.

At the behavioral level, PRL is evident in childhood, adolescence, and adulthood (Raab and Hartley, 2018). However, findings from studies using computational models of PRL to examine how value-based learning ability changes with age have provided mixed results. As reviewed by Nussenbaum and Hartley (2019), some developmental studies have reported that learning rates increase from childhood to adulthood (Davidow et al., 2016; Master et al., 2020) while others have reported that they do not change with age (Palminteri et al., 2016). Such mixed behavioral findings may reflect individual differences in one or all of the neurobiological processes underlying PRL (i.e., value representation associated with choices, updating of stimulus–reward outcome associations, shaping attention toward reward-predictive cues). Decades of neuroscience research have suggested that the development of PRL is supported by dopaminergic mesocorticolimbic brain pathways (reviewed in Daw and Tobler, 2014). DA neurons are involved in signaling reward prediction errors (i.e., discrepancies between expected and received rewards). When a reward is received after making a correct decision (e.g., winning), DA activity increases, which positively reinforces the same choice behavior that led to the reward (i.e., a win-stay strategy). Conversely, if the individual does not receive a reward, DA levels may decrease, which can negatively reinforce them to change their choice behavior (e.g., a lose-switch strategy). Thus, the Win-Stay-Lose-Switch (WSLS) strategy is a simplified, heuristic behavioral model of how the DA brain reward system adjusts actions in response to positive or negative reinforcement.

The WSLS models have been successfully applied to data from binary choice experiments with adults (e.g., Worthy et al., 2013; Worthy and Maddox, 2012; Worthy et al., 2012). Recent work has demonstrated that adding WSLS and reinforcement learning models lead to a better account of decision-making behavior from a wide variety of decision-making tasks with adults (e.g., Worthy and Maddox, 2014) and animals (e.g., Ohta et al., 2021) Also, individual differences in the value updating process are known to be well captured in learning rates (Nussenbaum and Hartley, 2019). Such rates reflect the degree to which reward feedback and reward prediction errors are incorporated into the updated value estimates during learning. However, existing studies in children have rarely considered individual learning rates or WSLS models in the investigation of key neural components of reinforcement learning. A number of studies using functional Magnetic Resonance Imaging (fMRI) have suggested that reward sensitivity is signaled by dopaminergic midbrain-fronto-striatal brain circuits in both adults (Averbeck and O’Doherty, 2022; Nasser et al., 2017; Schultz, 2016) and adolescents (Christakou et al., 2013; Cohen et al., 2010; Hauser et al., 2015). However, the temporal resolution of the blood oxygen level dependent signal has limited the ability of fMRI research to inform the potential cascade of unique neurobiological processes shaping learning on a trial-by-trial basis, which requires more precise temporal resolution.

The high temporal resolution of electroencephalogram (EEG) measures of brain activity makes such recordings particularly well-suited for studying the neural subprocesses associated with PRL. More specifically, the ability to extract and measure event-related potentials (ERPs) time-locked to stimulus and reward-feedback events provides a particularly effective opportunity to delineate the temporal cascade of neurocognitive processes related to reward processing across a trial (Amodio et al., 2014). To dissociate the specific neurocognitive processes supporting successful reinforcement learning, our prior work with college students (Van den Berg et al., 2019), as well as in other studies (e.g., Donaldson et al., 2016; Peterson et al., 2011; von Borries et al., 2013) has focused on three key neural components of reinforcement learning: (1) reward-feedback sensitivity, as measured by the frontal reward positivity (RewP), (2) later attentional modulation during feedback evaluation, the cental-parietal P300, and (3) rapid attentional shifting toward a favored stimulus using the N2pc.

The RewP ERP component is a frontal, positive-polarity brain wave that occurs 250–350 ms after gain feedback relative to loss feedback. With respect to RL theory (Holroyd and Coles, 2002; Sutton and Barto, 2018), the RewP is thought to index reward-related sensitivity in the mesocortical dopamine system, specifically reflecting reward prediction error signals that are sensitive to outcome valence and are larger for unexpected positive events relative to unexpected negative events (Holroyd and Coles, 2002). Although the RewP or its inverse, the Feedback-related Negativity, FN, calculated as loss-minus gain feedback in some earlier research (Krigolson, 2018) as a measure of response to reward has been widely investigated in adults (reviewed in Glazer et al., 2018; Kujawa, 2024), its role in PRL in children has not. Thus, we know little about how individual learning rates in preadolescents may modulate changes in the RewP amplitudes.

The RewP component is often followed by another feedback-evoked, later positive deflection, the P300 component. This component is a centro-parietally distributed wave that peaks between 400 and 600 ms after stimulus presentation (San Martín et al., 2013; Wu and Zhou, 2009). The P300 reflects general attentional processes involved in updating expectations based on unexpected or salient outcomes during evaluative processing (for reviews, see Glazer et al., 2018, San Martín, 2012) and has been used to investigate PRL with adults (e.g., Donaldson et al., 2016). Prior work has shown that the P300 reflects a longer-latency top-down-control process of outcome evaluation, suggesting that feedback information (e.g., probability and magnitude) in probability choice tasks guides future decisions for reward maximization in adults (San Martín et al., 2013; Wu and Zhou, 2009). Thus, if individuals receive unexpected loss feedback and decide to switch their choice on the next trial (thus employing a Lose-Switch strategy), the P300 amplitudes might be higher compared to when they received positive reward feedback. However, the functionality of the feedback-locked P300 (reward gain vs. loss feedback) during PRL in children has been little examined. More specifically, prior ERP studies reporting P300s in children have typically measured them during non-reward, cognitive processing, such as memory, attention, or executive function (reviewed in Peisch et al., 2021; Van Dinteren et al., 2014), or in reward-related feedback responses without consideration of reward contingencies (e.g., Carlson et al., 2009; Ferdinand et al., 2016; Yau et al., 2015). As a result, there is limited understanding of whether the P300 as measured in children during PRL will reflect attention allocation as a function of behavioral strategy.

As one learns which stimuli are more likely to generate reward outcomes, one tends to pay more attention to those stimuli. Such attention shifting toward reward-associated stimuli has been indexed by the N2-posterior-contralateral (N2pc) component, which typically emerges over posterior sites 180-to 300 msec following the stimulus in prior work with adults (Hickey et al., 2010; San Martín et al., 2016). Traditionally, the N2pc component has been extensively studied as an electrophysiological marker of selective visual attention in non-reward tasks (reviewed in Gupta et al., 2019; Zivony and Lamy, 2022). Recent studies, including our own (Van den Berg et al., 2019), suggest that N2pc amplitudes are modulated by reward expectations in adults (e.g., Chen and Wei, 2023; Taylor and Feldmann-Wüstefeld, 2024). Only a handful of ERP studies have examined the N2pc in children, either in the context of a reward task or more generally during visual search (Couperus and Quirk, 2015; Li et al., 2022; Shimi et al., 2014; Sun et al., 2018; Turoman et al., 2021) and/or working memory tasks (Rodríguez-Martínez et al., 2021; Shimi et al., 2015). Consistent with previous work in adults, prior studies with children indicate the N2pc is present during non-PRL attentional tasks, suggesting its potential as a marker of attentional selection during PRL in children as well (Couperus and Quirk, 2015; Li et al., 2022; Shimi et al., 2014; Sun et al., 2018; Turoman et al., 2021). However, such a possibility has not been directly tested. Accordingly, we know little about the functionality of the N2pc ERP component in children within a PRL framework – that is, whether it may reflect an index of early attentional shifting toward more reward-predicting stimuli in children, as has been shown previously in adults.

There were two main goals of the current study. First, we aimed to establish the feasibility and initial validation of a developmentally appropriate probabilistic reward learning task for preadolescent children, which we have termed the CPLearn task (Child Probability Learning task). Based on our results, we believe that this task would be a promising tool for studying both typical and atypical development of key neurocognitive processes underlying PRL. For example, alterations in attention (Keller et al., 2019) and impaired PRL (Chen et al., 2015) have been identified as key factors in hedonic functioning, which predict depression risk in children and adolescents (Keren et al., 2018; Morris et al., 2015; Saulnier et al., 2023). The design of the CPLearn task was based on our prior work investigating neural correlates of PRL processes in adults (van den Berg et al., 2019). Secondly, following prior theory (Hernstein et al., 2000), we examined key subprocesses of PRL in the context of behavioral learning strategies, namely, Win-Stay-Lose-Shift (WSLS). These subprocesses included: (1) reward-feedback sensitivity (i.e., RewP), (2) long-latency attentional processing of choice-outcome feedback (the P300) related to the WSLS strategy within the PRL framework, and (3) early attentional shifting toward high-reward stimuli (i.e., N2pc). We predicted that preadolescents who did not learn the stimulus-outcome associations very well (i.e., “poor” learners) would show smaller RewP amplitudes, consistent with the RL framework, compared to “good” learners, and/or would adopt less effective behavioral strategies. Finally, building on prior work showing that adults adopt effective strategies in later attention allocation in response to outcome feedback, as reflected in the P300 component (Donaldson et al., 2016; Von Borries et al., 2013), we hypothesized that the central-parietal, feedback-locked P300 would reflect modulation of reward value updating as a function of behavior strategy in preadolescent PRL learning (i.e., Win-Stay-Lose-Switch).

## Materials and methods

### Participants

Thirty children between 8 and 12 years old were recruited from a Research Participation Database at Duke University maintained by the Human Subjects Coordinator for the Department of Psychology and Neuroscience. In addition, methods included posting flyers on social media sites and distributing flyers to local schools were used to recruit participants (see Table 1 for Participants Demographics).

**Table 1:** Participant demographics.

	Mean ±SD
Age	9.94 years ±1.26
Income-to-needs ratio^1^	3 (±1)
	*n*
Biological sex at birth	
Females: males	15: 15
Race	
White	*n* = 24
Mixed racial background	*n* = 5
African American	*n* = 1
Asian	*n* = 0
Ethnicity	
Hispanic or Latino	*n* = 6
Not Hispanic or Latino	*n* = 24
Maternal education	
High school diploma	*n* = 1
Some college	*n* = 2
Graduated 4-year college	*n* = 10
Graduated from graduate or professional school	*n* = 17

^1There was n = 2 who reported “don’t know”. The Income-to-needs ratio was calculated by dividing total family income by a poverty threshold determined by the United States Census Bureau, which considered the year assessed and household family size. The income-need ratio is, by definition, 1.0 at the poverty line, and numbers above that are multiples of needs.^

Children who were color blind, reported a current and/or historical diagnosis of any psychiatric disorder, had a history of neurological disorder or insult (e.g., seizures, hydrocephalus, cerebral palsy, brain tumor, extended loss of consciousness, head trauma), were taking psychotropic medication for a mood or behavioral difficulty, or were left-handed were excluded from study participation. At the end of the EEG sessions, a $30 Amazon e-gift card was sent to the parents via email for the participation of their children in the study. Children also received candy and/or small toys after each completed session. Additional compensation was provided ($10 Amazon e-gift card per hour) to parents if testing sessions exceeded 2.5 h. This study was conducted in accordance with protocols approved by the Duke Institutional Review Board.

### Stimuli

The task stimuli consisted of face and house images that were used in our prior study with college students (van den Berg et al., 2019). These task stimuli were adopted from the Database of Faces^1^ (see Van den Berg et al. (2019) for more details).

### Task paradigms and procedure

Practice Runs: To implement an age-appropriate Probabilistic Reward Learning task, we conducted multiple behavioral pilot tests over an eight-month period. During this time, we adjusted the task instructions to ensure they were easy for children to understand. Also, we made sure that children were able to respond within a specified time window. All subjects performed two blocks of 18 trials each (i.e., 36 trials). The total completion time of practice sessions ranged from 29 s to 1,400 s, with a median of 656 s (i.e., approximately 11 min in total). The percentage of correct trials (i.e., choosing trials associated with more likely to get rewarded) for practice runs was 67.66% (*n* = 30).

### The child probabilistic reward learning task and localizer task

After two practice runs, participants completed the CPLearn take, followed by a face-vs-house neural-activation localizer task (for localizing the face-selective neural activity on the scalp and its timing), while EEG was recorded. The task was programmed and run on the OpenSesame platform for behavioral research (Mathôt et al., 2012), and event codes were sent to the EEG acquisition computer using the Python ‘egi’ package.

**Figure 1:**
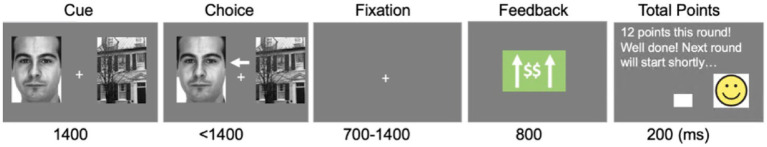
Child-version of probabilistic reward learning (C-Plearn) task. An example of one trial with reward feedback (i.e., green box in the fourth panel showing an upwards arrow and dollar signs).The task of the participants was to choose which stimulus type (houses of faces) was more associated with receiving reward points through trial-and-error feedback processing. If participants choose the non-set winner (i.e., the one not associated with getting reward points), they received loss feedback (i.e., red box including downwards arrow and dollar sign). The same task stimulus (i.e., face or house images) used in our prior work (Van den Berg et al., 2019) was employed, but we used different feedback stimulus: a green or red box along with an upward or downward arrow and dollar sign, as well as smiley vs. frown faces for reward and loss feedback, respectively.

CPLearn: The CPLearn task consisted of 20 blocks of 18 trials each, slightly fewer than the 20 trials per block in the original adult version (van den Berg et al., 2019) to accommodate reduced attention spans and lower tolerance for prolonged experimental tasks in children. Also, the probability of reward was set to 0.77 (instead of ranging randomly from 0.5 to 0.75 as in the original adult version) to mitigate frustration in the children and potential confusion noted during early piloting sessions using the original adult reward-probability levels. At the beginning of each trial, participants were presented with two images on the screen, on the left and right of fixation, one of a face and one of a house, and were instructed to choose between the two (see Figure 1 for an example of a trial). After the participants made their choice on each trial, they received feedback as to whether they won points or lost points on that trial. Participants were instructed to try to learn during each block whether faces or houses were more likely to result in winning points, and that winning more points would allow them to have more points for choosing at the end of the session small toys that had differing levels of cost. At the end of each 18-trial block, feedback was given as to how many points the participant had accrued up until that point.

Localizer task: Following the CPLearn task, participants completed a localizer task, which was originally designed to generate potential regions of interest for assessing the face-responsive sensory cortices in our prior work with adults (van den Berg et al., 2019). We used a very similar localizer task to that one used in our prior work with young adults (van den Berg et al., 2019), which consisted of 20 blocks of 10 trials each. During the task, participants saw single versions (not paired) on each trial of the same house and face images presented during the CPLearn task, but 20% were somewhat blurred, which served as infrequent targets, while the rest were clear (i.e., non-targets) (see van den Berg et al. (2019) for more details). The task of the participants was to press a button for any blurry images and indicate whether they were a face or a house by pressing a button. However, data from the localizer task was not of interest for the current study and was thus not included in our major analyses here. Rather, we just collapsed the data over the faces and houses, and focused on other questions for this study.

### Computation of reward-learning behavior strategies

To calculate each subject’s reward-learning behavior within the PRL framework, we defined win-stay (WS) trials as those in which the previous trial (*n* − 1) was rewarded (won) and the choice made on the current trial *n* was the same choice as that on trial *n* – 1 (i.e., “stayed” with the same choice of face or house made on the previous trial). We then calculated WS probabilities as the proportion of trials with WS behavior given a previously rewarded trial (i.e., after receiving gain feedback). We defined lose-shift (LS) trials as those in which the previous trial (*n* − 1) was not rewarded (i.e., receiving loss feedback) and the choice made on the current trial *n* differed from the choice on trial *n* − 1. We then calculated LS probabilities as a proportion of trials with LS behavior given that the previous trial was unrewarded (i.e., after receiving loss feedback) (see the formula below).


$$\mathrm{Proportion}\;\mathrm{of}\;\mathrm{Win}-\mathrm{Stay}\;\mathrm{trials}=\frac{\mathrm{Number}\;\mathrm{of}\;\mathrm{Win}-\mathrm{Stay}\;\mathrm{trials}}{N-1}$$

$$\mathrm{Proportion}\;\mathrm{of}\;\mathrm{Lose}-\mathrm{Switch}\;\mathrm{trials}=\frac{\mathrm{Number}\;\mathrm{of}\;\mathrm{Lose}-\mathrm{Switch}\;\mathrm{trials}}{N-1}$$

*N*-1, where *N* is the total number of trials, since the first trial does not have a previous trial to base a decision on.

### EEG data collection and processing

Continuous EEG was recorded using a 128-channel HydroCel Geodesic Sensor Net (Electrical Geodesics, Eugene, OR) and Net Amps 400 series amplifiers at a sampling rate of a 1,000 Hertz (Hz). Data was referenced online to the vertex (Cz) during acquisition, and impedances were maintained below 50 kilohms throughout the paradigm.

Offline EEG data preprocessing was performed using EEGLAB version 2021 (Delorme and Makeig, 2004) in MATLAB R2019b (The MathWorks Inc., Natick, MA) using custom scripts.^2^ According to the Maryland analysis of a developmental EEG pipeline recommended for pediatric populations (Debnath et al., 2020), we followed several key preprocessing steps as follows. First, twenty-four channels located on the outer ring of the sensor net were removed from the analyses. This preprocessing step was performed because participants were required to wear masks during the EEG sessions according to Duke COVID-related policy, which tended to impair sensor connectivity, and thus these channels had a particularly large number of artifacts during the recordings in the children. The data were then downsampled to 250 Hz, low-pass filtered at 40 Hz, and segments of data without relevant events (i.e., breaks) removed. Using the ERPLAB v8.2 plugin (Lopez-Calderon and Luck, 2014), a 0.1 to 30 Hz, 4th order Butterworth, bandpass filter was applied. The CleanLine plugin (Kappenman and Luck, 2012) was used to remove any remaining 60 Hz line noise (Mitra and Bokil, 2007). All data were re-referenced to the average of the two mastoids. Bad channels were removed by running the Clean Rawdata plugin: a channel was considered bad if (1) it was flat for more than five seconds, (2) contained more than four standard deviations of line noise relative to its signal, or (3) correlated at less than 0.8 to nearby channels.

According to current recommendations in the field (Debener et al., 2010; Debnath et al., 2020), a “copy” of the data was made, and then the following steps were applied (Delorme and Makeig, 2004): (1) the high-pass filter at a 1 Hz (4th order Butterworth), (2) Artifact Subspace Reconstruction (ASR; Mullen et al., 2015) with the burst criterion set to 20 (Chang et al., 2018) to remove large artifacts with Clean Rawdata, and (3) extended infomax Independent Component Analysis (ICA; Lee et al., 1999) with PCA dimension reduction (50 components). The resulting ICA matrix was then copied over to the original full-length data (i.e., the data just before the copy was made and a 1 Hz high-pass filter was applied). The ICLabel plugin (Pion-Tonachini et al., 2019) automatically removed independent components with a probability greater than 0.7 of being associated with an eye movements or blink. Application of the ICA matrix to the full-length data and subsequent removal of eye-related components allowed for the preservation of data that would have otherwise been removed by ASR or other artifact removal methods. Bad channels that were previously removed from the analyses were interpolated back into the data set using spherical splines (Perrin et al., 1989).

For the CPLearn task, epochs were extracted from 400 ms before until 800 ms after the onset of the relevant stimulus events (the choice cue-pairs or the feedback screens in the main task). All epochs were baseline corrected using the baseline period from-200 ms to the onset of the event. Artifact rejection using the TBT plugin (Trial-By-Trial basis; Ben-Shachar, 2018) removed epochs with at least 10 channels meeting the following criteria: (1) peak-to-peak amplitudes exceeding 100 μV within 200 ms windows sliding across the epoch by 20 ms increments, (2) voltages below −150 uV or greater than +150 μV, or (3) joint probabilities above three standard deviations for local and global thresholds. If less than 10 channels in an epoch met criteria for rejection, the epoch was not removed, but the identified channels were interpolated based on the surrounding channels for that epoch only. However, if there were more than 10 channels in the epoch that met criteria for rejection, the epoch was not included. Lastly, the averaged ERPs for each bin (i.e., Gain, Loss) for each subject were computed. Mean of the number of accepted Gain and Loss trials per subject were 184.3 and 128.2, respectively.

### Data binning and averaging

From the CPLearn runs, the epoched EEG data were binned by feedback (gains and losses) and choice (face or house), yielding the following average trial counts per subject: face gain [90.53 (± 24.97)], face loss [62.13 (±13.73)], house gain [93.76 (±19.60)], and house loss [66.06 (±14.76)], after excluding noisy epochs. As would be expected, there were significantly more gain trials than loss trials due to learning [*F* (1,29) = 43.52, *p* < 0.001]. On the other hand, there were no significant differences in the number of gain or loss trials for choosing a face vs. a house [*F* (1,29) = 0.46, *p* = 0.50].

### Analysis of ERPs evoked by the choice-cue pair

We calculated the N2pc response evoked by the choice-cue pair at the beginning of each trial to assess attentional orientation toward the two stimulus types as a function of what they will later choose that trial, as well as a function of what was the probability set winner for that block, using procedures similar to our previous work in adults (van den Berg et al., 2019). More specifically, the N2pc neural responses were assayed by the N2pc contralateral-vs-ipsilateral analysis typically calculated to derive this component (Luck, 2011), that is by subtracting the activity in the contralateral channels (relative to the chosen side) minus the ipsilateral channels and then collapsing over the left and right sides.

### Statistical analysis

For statistical analysis, we first calculated the time-locked ERPs as a function of the various event types and conditions. Based on previous literature, the reward-related Positivity (RewP) was measured from 275 to 375 ms following the feedback stimulus in a fronto-central ROI (E10, E11, and E16). The P300 was measured from 400 to 600 ms following the feedback stimulus in a central-parietal ROI (Left/Right: E37/E87, E31/E80, E53/E86, E60/E85, E67/E77, E61/E78, E54/E79; middle line: E129, E55, E62, E71) (Fischer and Ullsperger, 2013; San Martín, 2012; San Martín et al., 2013). Subsequently, the extracted values were analyzed with *t*-tests or repeated measures analyses of variance (ANOVA) to test for statistical significance (*p* < 0.05) with 95% confidence intervals, using SPSS version 2021.

The cue-related attentional bias, N2pc was measured from 175 to 225 ms after onset of the choice-cue image pair, from corresponding left and right occipital ROIs (E50/E101, E58/96; Hickey et al., 2010; Kappenman and Luck, 2012; San Martín et al., 2016). Subsequently, we calculated the difference in voltage contralateral vs. ipsilateral relative to the side on which the set-winner was presented, as well as contralateral vs. ipsilateral to the side the stimulus that would be chosen on that trial.

To calculate each subject’s individual behavioral learning rate, we used the same formula as in Van den Berg et al. (2019) and calculated on a trial-by-trial number basis employing a mixed-modeling approach using the *lme4* statistical package (Bates et al., 2015). A varying slope of condition per subject (the random effect) was included in the model if the Akaike Information Criterion (a measure of the quality of the model) improved. Statistical significance was set at *p* < 0.05, with the Satterthwaite’s degrees of freedom method as given by the *R* package *lmerTest* (Kuznetsova et al., 2017).

## Results

### Reward learning behavior

As mentioned above, to adjust the difficulty level of preadolescents (aged 8 to 12 years), the probability of reward in the CPLearn task was set to 0.77, which was slightly lower than the probability used in the original task with adults (Van den Berg et al., 2019). As presented in Table 2, participants showed approximately 64% correct choices (choosing the set winner – i.e., the stimulus associated with higher probability of being rewarded in a set) averaged across all the sets and trials. Mean reaction time (RT) for correct trials, calculated from the trials in which preadolescents chose the set-winner was 666.8 ms.

**Table 2:** Reward learning behavior data: reaction times for correct trials.

	Participants (*n* = 30)	Good learners (*n* = 15)	Poor learners (*n* = 15)	Statistics	Cohen’s d
% Correct trials	64.2%	72.8%	55.5%	*t* (28) = 4.73, *p* <0.001	0.1
Mean RT for correct trials (i.e., choosing set-winners)	666.7 ms (SD± 95.9 ms)	657.9 ms (SD± 64.4 ms)	675.6 ms (SD ± 121.3 ms)	*t* (28) = - 0.49, *p* =0.62	–

The child version of the Probabilistic Reward Learning Task consisted of twenty blocks, with 18 trials in each block. The probability of reward was set to 0.77 throughout the task. Correct trials were defined as those in which participants chose the stimulus associated with a higher probability of being rewarded.

### Trial-by-trial learning rates

**Figure 2:**
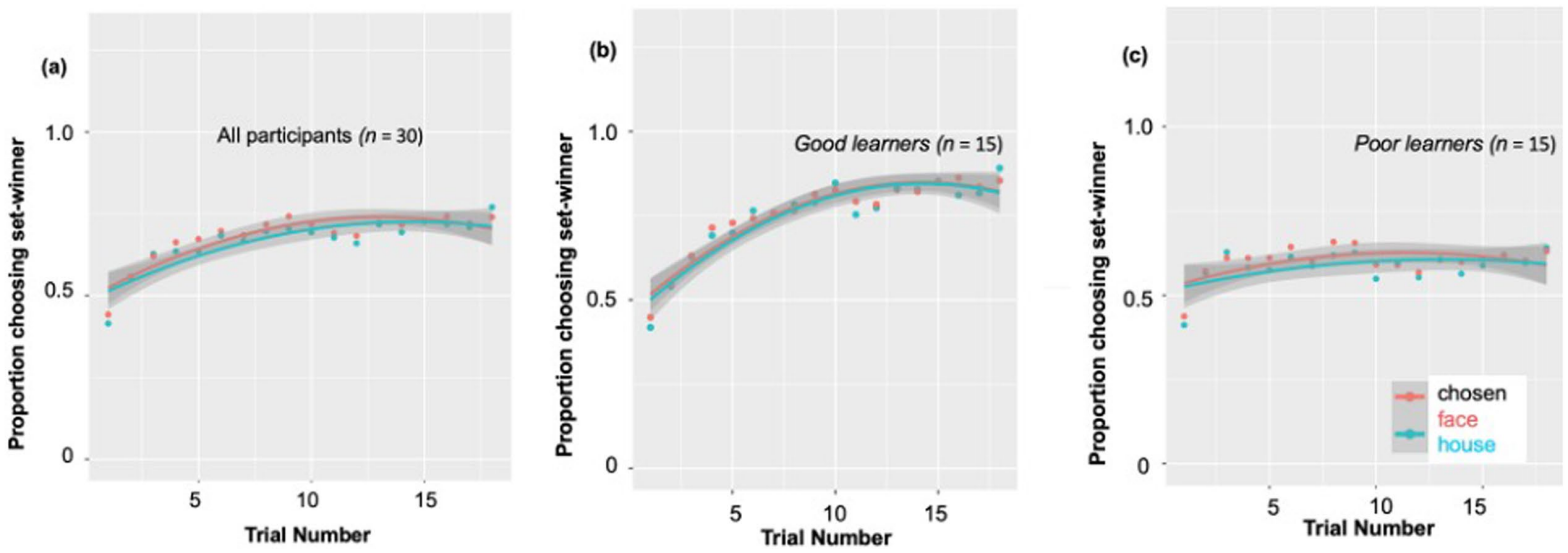
Individual differences in behavioral probabilistic reward learning. **(A)** All participants’ individual learning rates. During 18 blocks, the proportion of choosing set-winner (i.e., stimuli associated with a higher likelihood of being rewarded) for all participants ranged from 0.40 to 0.73. The individual learning curves revealed very large individual difference in reward learning, as the final proportion of choosing set winners in a particular block ranged from 0.26 to 1.0. Due to this large individual difference observed, we divided participants into good vs. poor learners according to a median split. **(B)** Learning rates for good learners showed a continuous increase from the first trial through the 9th trial to the last trial. **(C)** Learning rates for poor learners showed an increase from the first trial to the 9th trial, but no further increase during the remaining trials.

Consistent with our prior work in adults (Van den Berg et al., 2019), and as predicted, behavioral measures in the children demonstrated their reinforcement learning ability based on trial-by-trial reward-related feedback. According to a *post-hoc* paired *t*-test, the percent of choosing set winner on the last (18th) trial of the block (*M* ± *SD*: 0.75 ± 0.16) was significantly higher compared to on the first trial (*n* = 30, *M* ± *SD*: 0.42 ± 0.10) [*t* (29) = −9.54, *p* < 0.001, Cohen’s *d* = 2.47]. As presented in Figure 2A, at the beginning of each 18-trial set, participants chose the most likely winner for that set at only chance level (0.45–0.50), as would be expected. But by the end of the set, this proportion of choosing set-winner increased up to ~0.75 on average, indicating that the children had used the feedback across the trials of that set to learn to choose the more likely probability-based winner. Nevertheless, the individual learning curves revealed very large individual difference in this reward learning, as the final proportion of choosing set winners in a particular block ranged from 0.26 to 1.0 across subjects and blocks. Due to this large range, we wanted to explore whether ‘good’ versus ‘poor’ learners might show different patterns of reward-related ERP components, by dividing the subjects using a median split based on their learning rate (see Figure 2A).

Specifically, as shown in Figure 2B, learning rates for good learners showed continuous increases from the first trial (learning rate: 0.43) through the 9th trial (learning rate: 0.80) [*t* (14) = −9.57, *p* < 0.001, Cohen’s *d* = 5.11], to the last trial (learning rate: 0.87) [*t* (14) = 2.27, *p* = 0.03, Cohen’s *d* = 1.21]. On the other hand, learning rates for poor learners (Figure 2C) showed somewhat of an increase from the first trial (learning rate: 0.42) to the 9th trial (learning rate: 0.63) [*t* (14) = −3.77, *p* = 0.002, Cohen’s *d* = 2.01], but no increase during the last half of the trials [*t* (14) = −0.02, *p* = 0.98].

### Electrophysiological measures

#### The reward-related positivity amplitudes good vs. poor learners

**Figure 3:**
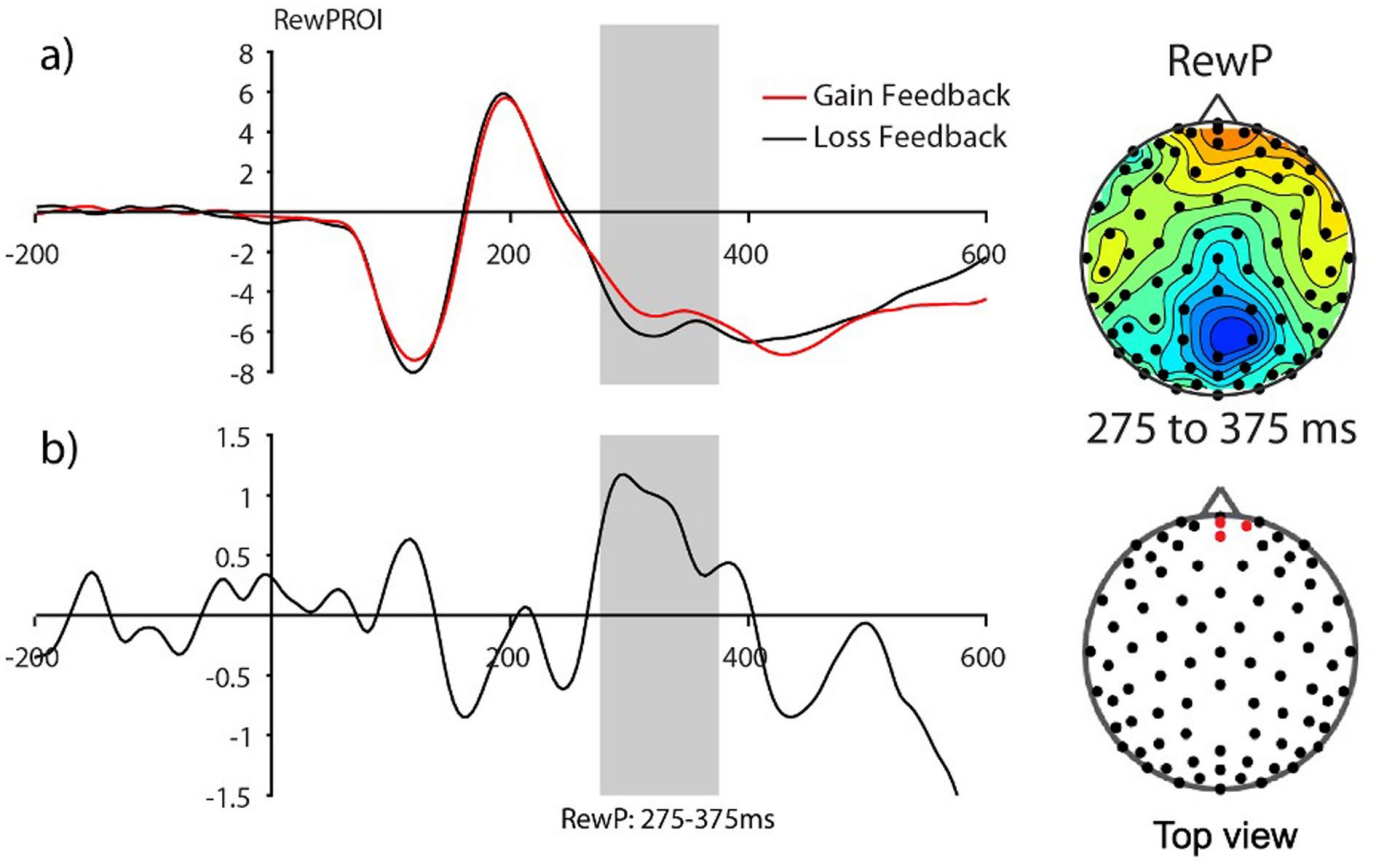
The Frontal reward-related potential (gain vs. loss feedback) in preadolescents (*n*=30). **(A)** Grand average feedback-cue evoked responses for Gain and Loss reward feedback from the fronto-central ROI (E10, E11, and E16) (see red dots in the topography for the location of the ROI). **(B)** The reward-related potential difference waveform during Gain minus Loss reward feedback. The reward-related potential was measured from 275 to 375 ms in a fronto-central ROI.

**Figure 4:**
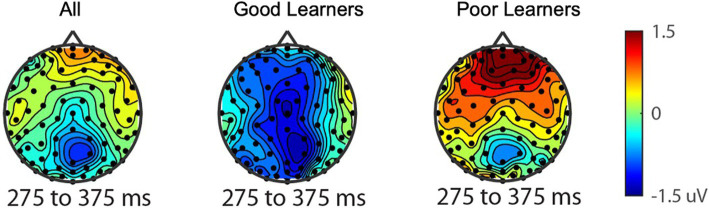
The reward-related positivity between good and poor learners. The comparison of the reward-related potential ERP marker measured from 275 to 375ms in the fronto-central ROI between good and poor learners. Dark red colored electrode sites in the frontal ROI shows significantly greater reward-related positivity amplitudes for poor learners (i.e., those with low learning rates) compared to good learners (i.e., those with high learning rates).

Consistent with prior work in adults, we anticipated the processing of gain vs. loss feedback would be reflected by the reward positivity wave, that is, the RewP in the fronto ROI, calculated from the gain-minus-loss difference waves. As presented in Figure 3, we found the presence of RewP around 275–375 ms after gain vs. loss feedback in our frontal ROI (E10, E11, E16) for young children. Given large individual differences in learning rates in our sample, we compared the RewP amplitudes between good vs. poor learners. In contrast to our expectation, the presence of the RewP amplitudes appear to be mostly driven by poor learners with lower learning rates. The independent *t*-tests revealed that poor learners had larger RewP amplitudes in the frontal ROIs compared to good learners [*t* (27) = −2.14, *p* = 0.04, Cohen’s *d* = 0.79] (see Figure 4).

#### The P300 during reward gain vs. loss feedback as a function of behavior strategy

**Figure 5:**
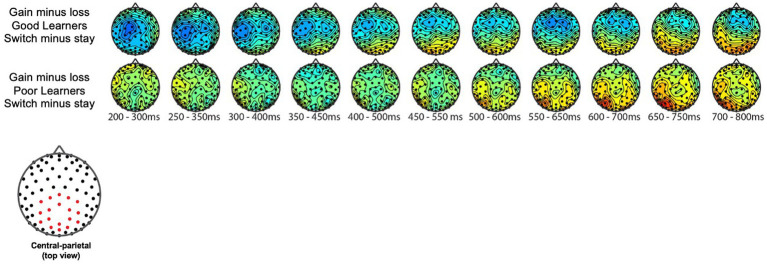
Topography of P300 during gain vs. loss feedback as a function of behavioral strategy. P300 amplitude differences in central and parietal ROIs on trials on which participants switched their responses on the next trial after gain feedback vs. after loss feedback. The red dot in the topography at the bottom shows the location of the central-parietal ROI. Top row: warm colored electrode sites indicate bigger P300 amplitudes on stay trials for good learners with high learning rates. The Bottom row: warm colored electrode sites indicate bigger P300 on stay trials for poor learners with low learning rates.

**Figure 6:**
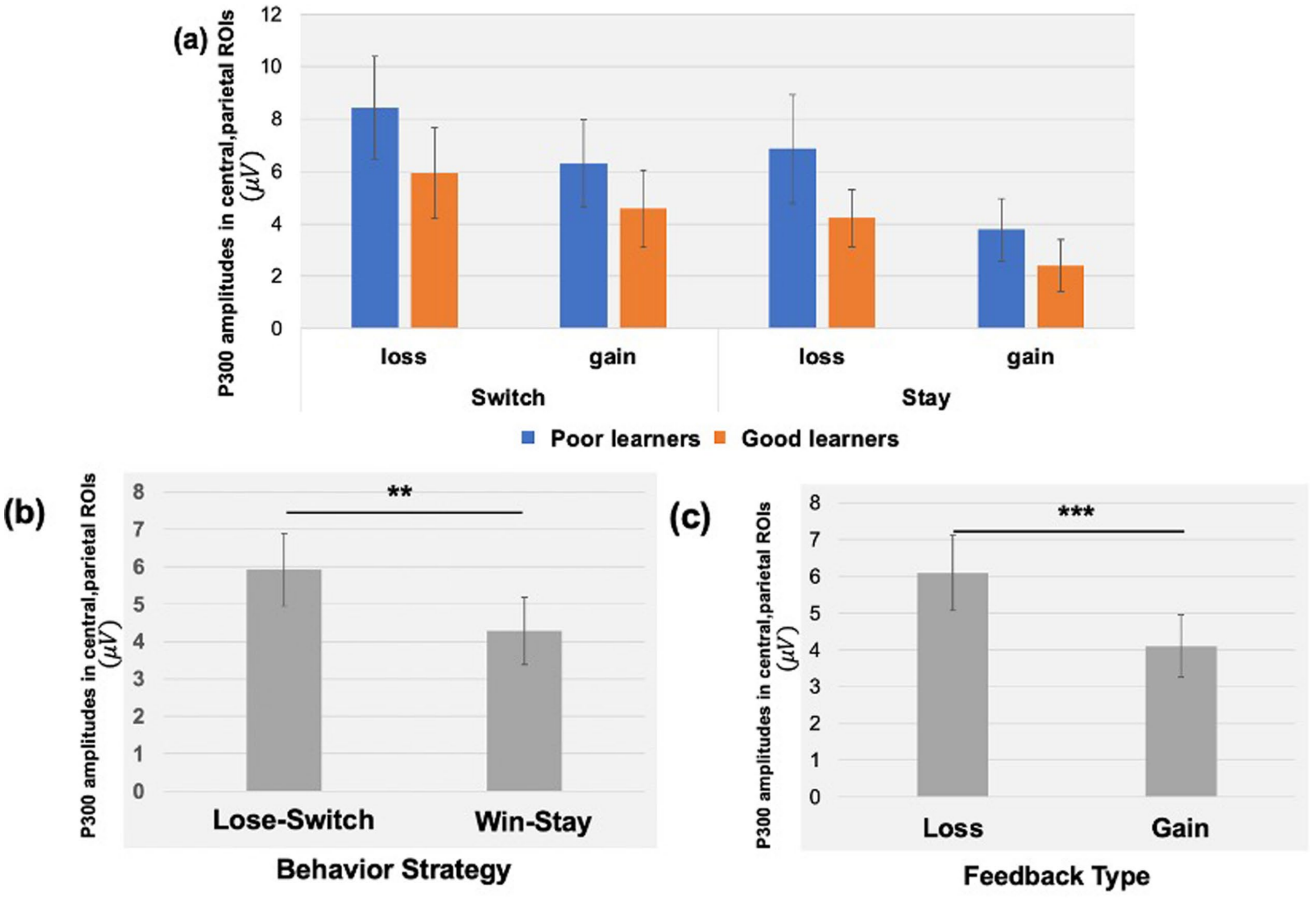
P300 amplitudes in central, parietal ROIs as a function of feedback type and behavioral strategy. **(A)** Using P300 amplitudes in the central -parietal ROIs between 400 and 600 ms for good and poor learners, we found that poor learners (i.e., those with low learning rates), relative to good learners, showed higher P300 amplitude differences in central-parietal ROI when they switched their responses on the next trial after gain feedback vs. after loss feedback. **(B)** For all children, we found greater P300 amplitudes in the central-parietal ROI when participants switched their choice on the next trial after loss feedback (i.e., lose-switch strategy) compared to when they chose the same choice after gain feedback (i.e., win-stay strategy). **(C)** The P300 amplitudes in the same central-parietal ROI were higher when participants received loss feedback compared to gain feedback. Error bars indicate standard error; asterisks indicate significance level: **p = 0.01; ***p =.005.

We examined the updating of reward values as a function of reward learning, as reflected by modulation of the P300 as a function of behavioral strategy. Figure 5 shows the P300 in response to gain vs. loss feedback as a function of trial-to-trial strategy (i.e., Switch vs. Stay) between good and poor learners. Using P300 amplitudes in the central ROIs between 400 and 600 ms for good and poor learners, we conducted a repeated-measures ANOVA with two within-subject factors (i.e., behavior strategy: switch versus stay, feedback: gain vs. loss) and one between-subject factor (i.e., good vs. poor learners). The ANOVAs were conducted to examine whether poor learners, relative to good learners, showed greater P300 amplitudes in response to gain vs. loss feedback as a function of switch behavior strategy (see Table 3 for repeated measures ANOVA results). As shown in Figure 5, poor learners (i.e., those with low learning rates), relative to good learners, showed higher P300 amplitude differences in a central-parietal ROI on trials when they changed their responses on the next trial after gain feedback vs. after loss feedback. The repeated ANOVA analysis revealed no main effect on the P300 of learner type [poor vs. good: *F* (1, 27) = 1.45, *p* = 0.23, $\eta_{p}^{2}$ : 0.05] (see Figure 6A). We did observe, however, main effects of behavior strategy with the greater P300 amplitudes on lose-switch trials than win-stay trials [*F* (1, 27) = 9.11, *p* = 0.005, $\eta_{p}^{2}$ : 0.25] and feedback with greater P300 amplitudes on loss compared to gain trials [*F* (1, 27) = 7.00, *p* = 0.01, $\eta_{p}^{2}$ : 0.21], as presented in Figures 6B,C, respectively, but no interaction of feedback by behavior strategy [*F* (1, 27) = 0.34, *p* = 0.56, $\eta_{p}^{2}$ : 0.01] or of behavior strategy by learner type [*F* (1, 27) = 0.38, *p* = 0.54, $\eta_{p}^{2}$ : 0.01] or feedback by behavior strategy by learner type [*F* (1, 27) = 0.09, *p* = 0.76, $\eta_{p}^{2}$ : 0.003] (see Figure 6). According to a *post-hoc* paired *t*-test, however, after receiving gain vs. loss feedback, children showed greater P300 amplitude differences in the central-parietal ROI when they switched their choice on the subsequent trial compared to when they chose the same response [*t* (28) = 2.68, *p* = 0.012, Cohen’s *d* = 0.32] (see Figure 6B). Also, P300 amplitudes in the same central-parietal ROI were higher when children received loss feedback compared to gain feedback, collapsed across both good and poor learners [*t* (28) = 3.03, *p* = 0.005, Cohen’s *d* = 0.39] (see Figure 6C), consistent with adult findings (Van den Berg et al., 2019).

**Table 3:** Results of repeated measurements analysis of variance (ANOVA) on amplitudes of P300 component.

P300 amplitude	*F* (1,28)	*p*	$\eta_{p}^{2}$
Behavior strategy (i.e., win-stay, lose-switch)	9.11	0.005*	0.25
Learner type (i.e., good versus poor learners)	1.45	0.23	0.05
Feedback type (i.e., gain, loss)	7.00	0.01*	0.21
Feedback × behavior strategy	0.34	0.56	0.01
Behavior strategy × learner type	0.38	0.54	0.01
Feedback × behavior strategy × learner type	0.09	0.76	0.03

Asterisks indicate significant effects. To examine how behavioral strategies influence feedback-driven P300 amplitudes based on learner type, a repeated measures ANOVA was conducted with behavioral strategies (i.e., Win-Stay, Lose-Switch), feedback types (i.e., gain, loss) as within-subject factors, and learner type (i.e., good versus poor learners) as a between-subject factor.

#### The N2pc component reflects attentional bias toward choice behavior

**Figure 7:**
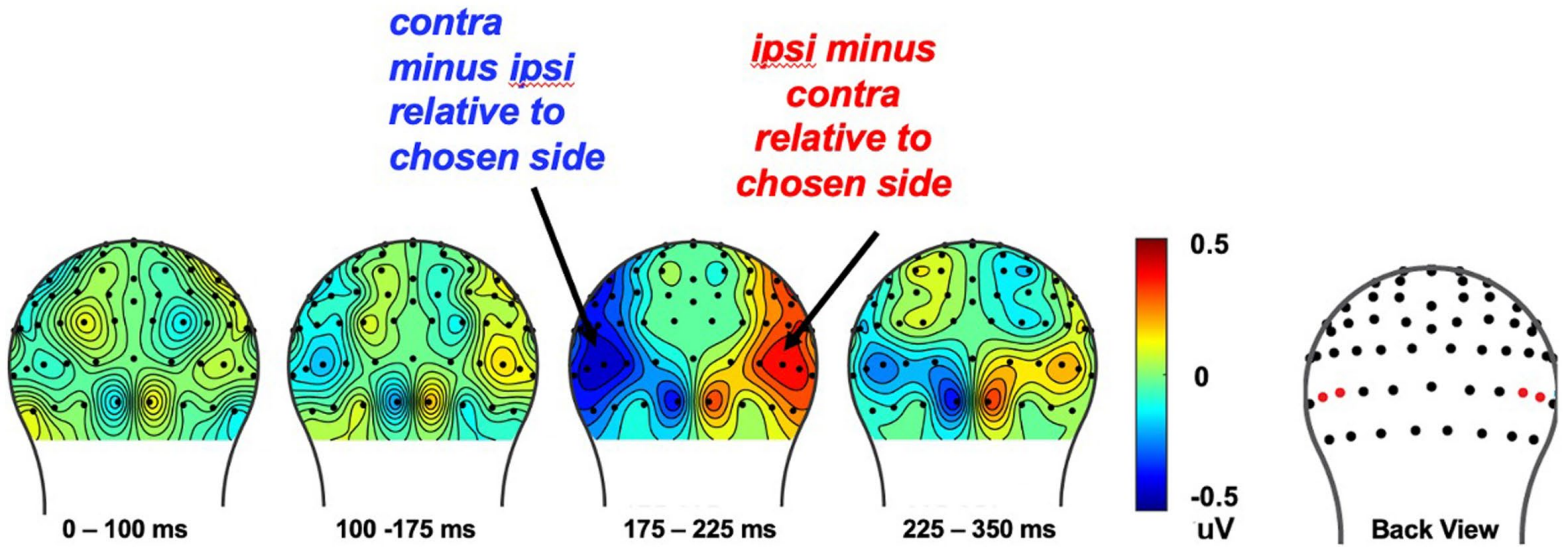
The cue-evoked attentional bias of N2pc toward the to-be-chosen side on each trial. To increase signal-to-noise ratio, we collapsed over whether the chosen image was that of a face or a house and analyzed the effect of choice behavior on the N2pc over the course of the 18-trial set. The cue-evoked N2pc amplitudes measured after onset of the cue image pair, from corresponding left and right occipital ROIs was found from 175 to 225ms (see red dots in the topography for the location of the ROI electrodes).

In our prior work in college students (Van den Berg et al., 2019), we found that the N2pc changed across the block for the set winner as that set winner was learned, as well as a function of the stimulus that would be chosen on that trial. In the current study, however, we did not find an effect for the set winner, but only for the behavioral choice regardless of whether or not reward learning had been consolidated. More specifically, we first analyzed the effect of choice on the N2pc over the course of the 18-trial set by collapsing over whether the chosen image was that of a face or a house (to increase the signal-to-noise ratio). Then, we analyzed the relationship between the N2pc elicited by the cue-pair stimulus relative to the behavioral choice made later in the trial, regardless of what the set-winner was. This analysis showed the presence of an N2pc for the to-be-chosen stimulus type in the latency of 175–225 ms after the onset of each cue-pair stimulus (E50/E58) [*t* (29) = −2.85, *p* < 0.008] (see Figure 7). These results indicate that children shifted their attention to the stimulus type that they were going to choose that trial. However, we did not find a significant N2pc for the set winner in the choice-pair cue for all subjects [*t* (29) = −0.17, *p* = 0.86]. Lastly, independent *t*-tests on the N2pc amplitudes as a function of the set winner, measured in the ROIs from 175 to 275 ms between good and poor learners, revealed no statistical differences [*t* (28) = −1.76, *p* = 0.09], even though the behavior indicated that the good learners were learning the reward associations better.

#### The interaction between learning rates and behavioral strategies

**Figure 8:**
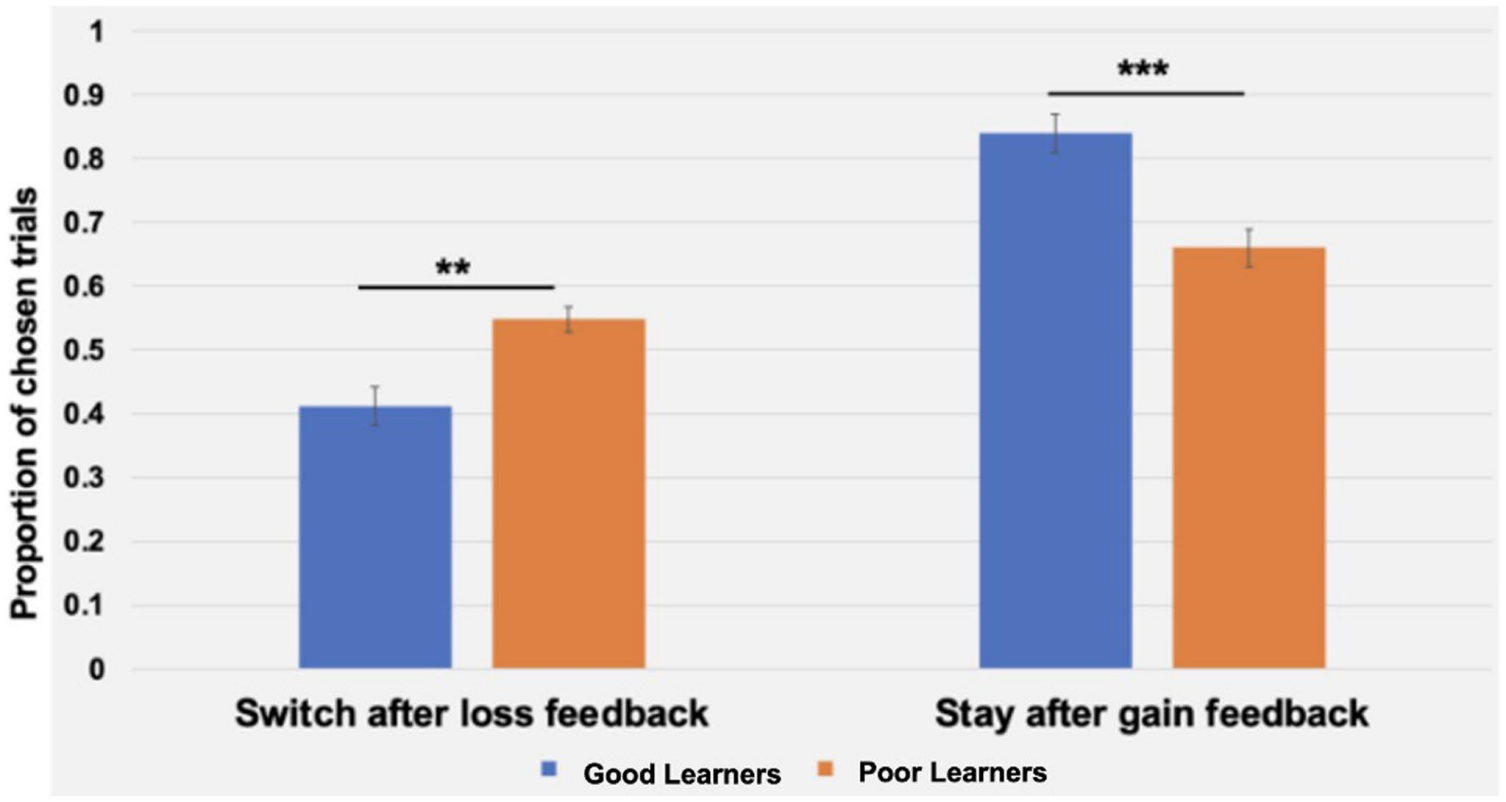
The interaction of behavioral strategies and learning types during the Child-version of Probabilistic Reward Learning task (CPLearn). During 18 blocks of the task, poor learners with low learning rates were more likely to change their choice on the next trial following loss feedback (i.e., Lose-Shift strategy). On the other hand, good learners with high learning rates were more like to make the same choice on the next trial following gain feedback (i.e., Win-Stay strategy). The error bars indicate standard error. **p = 0.007; ***p <0.001.

Beyond replicating the presence of three ERP components in preadolescents, as described above, we expanded on prior work with children by examining the temporal dynamics of behavioral strategies and learner types. To this end, a repeated measures analysis of variance (ANOVA) was conducted with behavioral strategies (i.e., WS, LS), blocks (i.e., 1–10 vs. 11–20 blocks) as within-subject factors, and learner types (i.e., good versus poor learners) as a between-subject factor. As presented in Table 4, there were main effects of strategy (*p* < 0.001) and a significant interaction of strategy and learner types (*p* < 0.001) but no main effects of block half and learner types or interaction effects of block by learner types or strategy by block by learner types. According to *post-hoc t*-tests to identify the sources of interaction effect of learner type and strategies, good learners were more likely to choose the same stimulus type (i.e., Win-Stay, WS behavior strategy) on the trial after one with a gain feedback [*t* (28) = 3.92, *p* < 0.001, Cohen’s *d* = 1.43]. In contrast, poor learners were more likely to change their response choice (i.e., Lose-Shift, LS behavior strategy) on the next trial after loss feedbacks [*t* (28) = −2.89, *p* = 0.007, Cohen’s *d* = 1.05] (see Figure 8).

**Table 4:** Results of repeated measures analysis of variance (ANOVA) on reward learning behavior and strategies.

Reward learning behavior	*F* (1,28)	*p*	$\eta_{p}^{2}$
Strategy (i.e., win-stay, lose-switch)	58.75	2.3806E-8***	0.67
Blocks (i.e., 1-10, 11-20 blocks)	4.04	0.05	0.12
Learner type (i.e., good learners with high learning rate, poor learners with low learning rate)	0.51	0.47	0.01
Strategy × learner type	19.23	0.000148***	0.41
Block × learner type	3.24	0.08	0.10
Strategy × block	4.07	0.05	0.12
Strategy × block × learner type	0.43	0.51	0.02

***p < 0.001. To examine differences in behavioral strategies between good and poor learners throughout the CPLearn task, a repeated measures ANOVA was conducted with behavioral strategies (i.e., Win-Stay, Lose-Switch), blocks (i.e., 1-10 vs. 11-20 blocks) as within-subject factors, and subgroups (i.e., good versus poor learners) as a between-subject factor.

**Figure 9:**
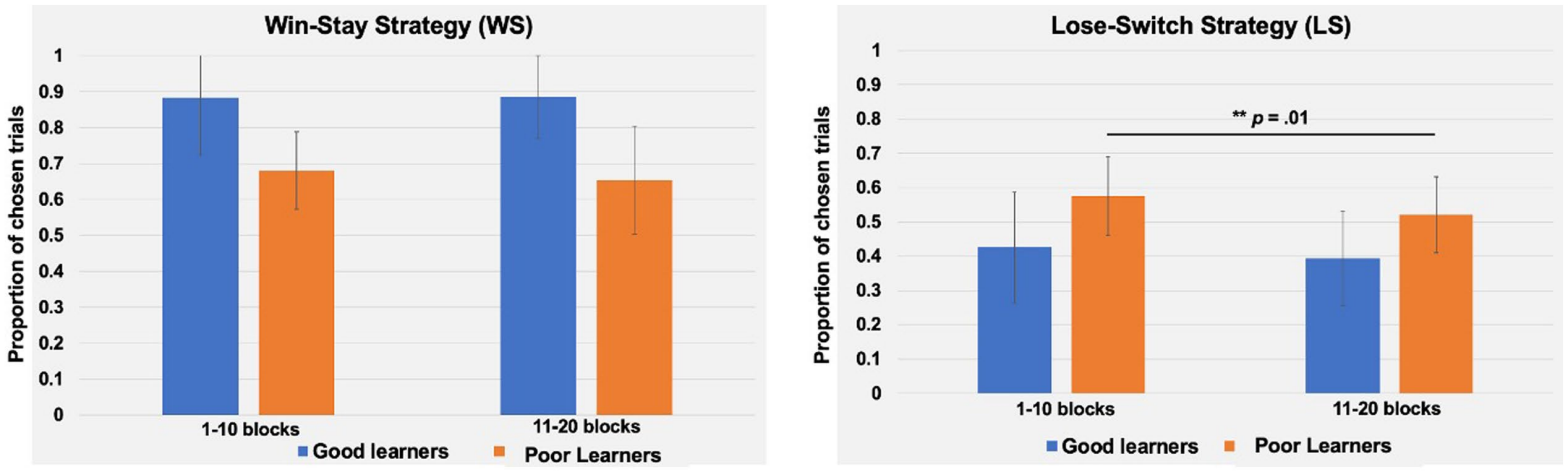
Comparison of behavioral strategies between good and poor learners in the first-half and second-half blocks. The win-stay strategy is defined as the proportion of trials in which subjects choose the same option as the previous trial, provided that previous trial was rewarded. The lose-switch strategy is defined as the proportion of trials in which subjects choose a different stimulus/option from the previous trial, provided that the previous trial was unrewarded. The proportion of win-stay and lose-switch strategy trials did not differ from the first to second half of the task for good learners (*n* =15). However, for poor learners (*n* =15), the proportion of lose-switch strategy trials was lower during the second half of the task, compared to the first half.

Given the trend-level main effects of blocks (*p* = 0.05) and the interaction between strategy and block (*p* = 0.05) or block and learner types (*p* = 0.08), additional *post-hoc* paired *t*-tests were conducted to identify potential temporal changes in behavioral strategies depending on learning type throughout the task. As presented in Figure 8, good learners’ proportion of LS in the first-half blocks (mean ± SD: 0.42 ± 0.16) vs. in the second-half blocks (mean ± SD: 0.39 ± 0.13) did not differ [*t* (14) = 1.59, *p* = 0.13]. Similarly, good learners’ proportion of WS trials in the first-half (mean ± SD: 0.83 ± 0.15) vs. in the second-half blocks (mean ± SD: 0.86 ± 0.11) did not change through the task [*t* (14) = −1.25, *p* = 0.23]. On the other hand, poor learners’ proportion of LS in the first-half block was higher (mean ± SD: 0.57 ± 0.11) compared to one in the second-half blocks (mean ± SD: 0.52 ± 0.11) [*t* (14) = 3.00, *p* = 0.01, Cohen’s *d* = 0.45] while proportion of WS in the first-versus second-half blocks were not different [*t* (14) = 1.00, *p* = 0.33] (Figure 9).

## Discussion

Consistent with our prior work in adults (Van den Berg et al., 2019), our behavioral CPLearn findings in children indicate that, at least at a group level, preadolescents can learn which stimulus type in a pair (here a face vs. a house) is more likely to lead to a higher probability of reward over a series of trials. At the neural level, we found modulation of key neural components of reinforcement learning for preadolescents: (1) RewP, (2) P300 and (3) N2pc components. Specifically, poor learners (i.e., those with low learning rates) showed greater reward-related positivity amplitudes relative to good learners (i.e., those with high learning rates), indicating greater reward sensitivity. As expected, P300 amplitudes during feedback evaluation were modulated by different behavior strategies. We also found attention shifting toward to-be-chosen stimuli, as evidenced by cue-evoked N2pc amplitudes, but not toward the set winner. These results suggest that the CPLearn task is a behaviorally-validated one, which could be a promising tool in preadolescents to identify either or both typical and atypical RL-related specific neurocognitive processes (i.e., reward sensitivity, attention shifting toward favored stimuli, and/or updating reward values according to feedback).

### Reward positivity amplitude varies based on learner types

Most existing ERP studies with children (age from 8 to 12 years) have focused mainly on the RewP component as a neural index of reward responsivity by comparing reward vs. no-reward feedback processing (e.g., Burani et al., 2019; Ethridge et al., 2017; Ferdinand et al., 2016; Kessel et al., 2016; Kessel et al., 2019; Lukie et al., 2014). Consistent with this prior work, there was a small-amplitude RewP positivity averaged across all preadolescents. In contrast to our predictions, however, and providing a unique extension of this prior work, we found that poor preadolescent learners with low learning rates showed greater amplitudes of the RewP components than did good learners, indicating greater reward-feedback sensitivity. Indeed, good learners actually appeared to show an inverted (negative-polarity) difference for gain minus loss.

Based on many animal and human studies, the basal ganglia are suggested to evaluate ongoing events in the environment and predict whether the events will more likely lead to success or failure. When the basal ganglia revise the predictions for the better, a phasic increase in the activity of midbrain dopaminergic neurons occurs. But, once the stimulus–reward outcome associations have been learned, there is a phasic decrease in the activity of midbrain dopaminergic neurons during reward delivery, specifically when reward-predictive cues are presented (reviewed in Daw and Tobler, 2014). For example, according to seminal animal work by Schultz et al. (1997), when a reward was consistently paired with a predictive stimulus, the phasic increase in dopamine firing rate observed at the time of reward delivery *diminished over time* and instead a phasic increase in dopamine firing rate was observed shortly after the onset of the predictive stimulus. More recently, this has also been demonstrated in humans. According to the Krigolson et al. (2014) study with adults (*n* = 18), novel rewards elicited neural responses at the time of reward outcomes that *rapidly decreased in amplitude with learning*. Furthermore, they identified the presence of the RewP component at choice presentation, a previously unreported ERP that has a similar timing and topography (Krigolson et al., 2014).

In line with the RL framework (Holroyd and Coles, 2008), we speculate that the blunted (or even inverted) RewP effect that we observed in good learners with high learning rates in response to gain-minus-loss outcomes might reflect such phasic decrease in dopamine firing associated with learning, but it might be the case that these subjects had RewP increases in response to the reward-predicting cues instead. In contrast, poor learners with low learning rates might have showed greater RewP during the reward outcome period, as they had not learned the stimulus-outcome associations. Nevertheless, while the current study is unable to inform this distinction given the absence of post training trials (i.e., measuring ERPs in response to isolated cues following training), it would be of value for future work to explore this possibility.

### Behavioral strategies are reflected by feedback-locked P300 amplitudes in preadolescents

The P300 ERP component has been widely used for evaluating cognitive processes related to evaluating unexpected or salient information such as loss feedback and adapting behavior. Despite successful application of P300 ERP component to investigate reward-based decision-making in adults (e.g., Schuermann et al., 2012; San Martín et al., 2013), prior studies with children have typically measured the P300 component during non-reward, cognitive processes such as memory, attention or executive function and without consideration of a reinforcement learning framework (reviewed in Peisch et al., 2021; Van Dinteren et al., 2014).

In the current study, and in line with previously reported findings in adults, we provide novel findings in preadolescent children demonstrating the contextual modulation of the feedback-locked P300 over the parietal cortex during reinforcement learning. More specifically, P300 amplitudes were larger following loss feedback rather than gain feedback in both good and poor learners. As such, our current findings are in line with prior work suggesting that the P300 component is typically elicited by the presentation of salient information (e.g., unexpected loss feedback) during the feedback evaluation process (reviewed in Glazer et al., 2018). Further implications of the P300 in relation to behavioral strategies (i.e., Win-Stay-Lose-Switch; WSLS) are discussed below.

The WSLS strategy model in the current study revealed that poor preadolescent learners tended to more automatically “switch” their choice following losing feedback, a finding consistent with prior work with college students (Worthy and Maddox, 2014). The WSLS is a rule-based strategy that has been shown to be commonly used in binary outcome choice tasks (e.g., Otto et al., 2010). According to Worthy and Maddox (2014), college students with higher probabilities of shifting following a loss trial (i.e., “Lose-Shift” behavior strategy) showed poorer learning performance. Along with prior work with adults, our current findings add converging evidence that combining the WSLS and PRL models may provide a better account of decision-making behavior even in preadolescents compared to a single PRL model. Also, it is possible that preadolescents with greater reward sensitivity, as reflected by greater RewP amplitude, might have been more distracted when exposed to cues that were previously associated with high reward values but were associated with non-reward outcomes. Such distraction might have led to choosing inefficient learning strategies, such as the Lose-Shift strategy. Indeed, a recent meta-analytic review by Rusz et al. (2020) showed converging evidence for strong reward-driven distraction effects on reward-learning mechanisms.

Consistent with prior work with college students (van den Berg et al., 2019), preadolescents learned the stimulus–reward outcome association at a group level. Within the PRL framework, our behavior data indicated major differences in strategy between good and poor learners, with good learners using win-stay strategy and poor learners using more of a lose-switch strategy. PRL theory generally assumes that the most recent outcomes exert the most influence on the current choice, such that behavior on a given trial, *n*, depends particularly upon the choice and outcome on the preceding trial, *n* − 1. This relationship has been formalized as a simple, heuristic learning strategy called Win-Stay-Lose-Shift (WSLS: Hernstein et al., 2000). In our prior work in college students (San Martín et al., 2013), the amplitude of the feedback-locked P300 over central and parietal cortex, but not of the FN (RewP), predicted behavioral adjustments (i.e., lose-switching strategy) on the next trial. These results are in line with the more general context-updating hypothesis of the P300 ERP component (Nieuwenhuis et al., 2005). According to this view, decisions on each trial are informed by an internal model of the symbol/probability (win) contingencies, and the P300 amplitude reflects the extent of the feedback-triggered revision of such a model. Thus, the feedback-locked P300 distributed over central and parietal cortex may reflect adjustment processes to maximize gain and/or minimize loss in the future. In line with this view, the present study also provided novel findings showing the modulation of the P300 over central and parietal cortex as a function of behavioral strategy and feedback type, in that the feedback-locked P300 amplitudes were greater on lose-switching trials compared to win-staying trials, and in response to loss vs. gain reward feedback. In contrast to our expectation, however, we did not find overall differences in the feedback-locked central-parietal P300 amplitudes between good and poor learners, even though our data indicated a modulation of the P300 amplitudes as a function of behavioral strategy and feedback more generally. We cannot rule out a possibility that there was not enough power to detect the interaction effect of P300 amplitudes and learner types due to limited number of participants (*n* = 15 per each learner type).

### N2pc ERP component indexes early attention shifting toward the to-be-chosen stimulus

One key component of PRL processing is the instantiation of attentional shifts toward stimuli associated with higher probability of reward. Previously learned reward values can have a pronounced effect on the allocation of selective attention, which can be indexed as an increase in amplitude of the attention-sensitive, lateralized negative deflection (the N2pc) contralateral to the set-winner in the cue-pair presentation. Prior ERP studies in adults have reported that once a stimulus–reward association is learned, the presentation of that stimulus tends to trigger a larger or stronger attentional shift. Additionally, this learned association can help predict the likelihood of the choice that will be made on that trial (Hickey et al., 2010; Hickey and van Zoest, 2012; San Martín et al., 2016; Van den Berg et al., 2019).

Consistent with our prior work with adults (Van den Berg et al., 2019), we also observed the presence of an N2pc for the to-be-chosen stimulus type after the onset of each cue-pair stimulus. These N2pc changes indicate rapid orientation of spatial attention toward the to-be-chosen stimulus on that trial, regardless of prior history of reward outcome for the set-winner across blocks. However, we did not observe an increased N2pc toward the set winner stimulus type that was being learned across the block, an effect previously observed in young adults (van den Berg et al., 2019). We speculate that one possibility for this lack of effect was that any learning-based modulation of the attention-sensitive N2pc amplitudes may have been too small to be detected here. This may have been because it occurred only in the good learners, was relatively small in magnitude, and occurred only during the later trials of the blocks. Such an idea should be tested in future studies with larger samples of both good and poor child learners.

The N2pc is a cue-evoked component that is often observed in both children and adults typically involving visual attention, when participants are required to shift spatial attention to potential target items. It is thought to indicate attentional-control and target-detection systems driven by parietal and frontal cortices (reviewed in Luck, 2011). The fact that we observed an N2pc for the to-be-chosen stimulus on a trial shows that these children were able to shift their spatial attention and that this shift was reflected neurally by an N2pc. We speculate, however, that this attentional shifting process might not have been able to be linked very well to the reward learning and updating function in these children. According to source location studies of the N2pc component in adults, it is thought to be composed of two distinct neural responses - an early parietal source (180-200 ms) and a later occipital-temporal source (220-240 ms) (e.g., Hopf et al., 2000). The later occipital-temporal cortex may play a particularly important role in RL by integrating visual information with reward expectations, allowing for the identification and learning of relevant visual cues (i.e., set winner in current study) that predict positive or negative outcomes (Tomov et al., 2023). In the current study, the absence of modulation in N2pc amplitudes toward the set-winner stimulus in preadolescents may suggest that these children have not yet developed their ability to engage the additional resources associated with linking visual input with updated reward value, which are thought to come from the later-developing temporal-occipital cortex and/or frontal cortex (Tomov et al., 2023). Future source localization studies of the N2pc, with direct comparisons of preadolescents, adolescents, and adults, are needed to clarify the neural generators of the N2pc component in a developmental context.

## Limitations and future directions

As with all research, the current study should be viewed within the context of its limitations. This includes having a relatively small sample size for examining individual level differences and differential subgroup interactions, which may have resulted in our missing more nuanced distinctions in our behavioral measures of RL behavior and brain function (e.g., no significant N2pc to the stimulus with a higher likelihood of reward – i.e., the set winner – as that reward association was being learned). In addition, our sample was limited to preadolescent children, without a direct comparison to other age groups, such as adolescents. Future studies could thus replicate the current findings with the inclusion of different age groups. Furthermore, most participants in the current study were white, preadolescents from fairly affluent parents. Emerging evidence suggests that lower socioeconomic status (SES) may influence reward sensitivity and learning behavior differently than high SES (Decker et al., 2024). Therefore, the inclusion of children and adolescents from diverse SES background will be important for future studies using RL paradigms. Importantly, given the lack of ERP studies in children and adolescents examining attentional modulation in the context of reward learning, future studies should address how early versus late attention processing (e.g., reflected by the N2pc and P300, respectively) underlie reward sensitivity by comparing cue-driven versus feedback-driven RewP components.

## Conclusion

In summary, the results of the current study provide novel insights into core neurocognitive processes associated with preadolescents’ behavior during a child-appropriate probabilistic reward learning task: (1) The RewP component was modulated by individual learning rates in preadolescents: good learners with high learning rate appear to show more blunted (or even inverted) RewP amplitudes with their learning; (2) There was evidence of an updating of reward values as a function of behavior strategy, reflected by the modulation of the feedback-elicited P300 amplitudes depending on lose-switch vs. win-stay strategies in the good vs. poor learning groups; and (3) There was an early attentional orienting toward the stimulus that would be chosen on a trial, as evidenced by the N2pc, but such an orienting process was not observed as a function of the learning of the reward associations. Thus, the current study provides initial validation for the CPLearn task as a promising tool for investigating the relationships between attentional processing, learning, and reward sensitivity in preadolescents, while also providing some initial findings about the neural underpinnings of these cognitive functions. This line of future research, including longitudinal studies that include both children and adolescents, could reveal developmental changes in neural maturation and behavioral strategies underlying the development of effective reward learning abilities. In addition, the present work may help advance our understanding of the neurobiological factors contributing to the risk of depression and other cognition-development issues in this key demographic group.

## Data Availability

The datasets and code used in this study are available upon reasonable request. Requests to access the datasets should be directed to ychung@kean.edu.

## Footnotes

^1^ https://www.cl.cam.ac.uk/research/dtg/attarchive/facedatabase.html

^2^codes are available at: https://github.com/gaffreylab
